# Evidence of a Vocalic Proto-System in the Baboon (*Papio papio*) Suggests Pre-Hominin Speech Precursors

**DOI:** 10.1371/journal.pone.0169321

**Published:** 2017-01-11

**Authors:** Louis-Jean Boë, Frédéric Berthommier, Thierry Legou, Guillaume Captier, Caralyn Kemp, Thomas R. Sawallis, Yannick Becker, Arnaud Rey, Joël Fagot

**Affiliations:** 1 GIPSA-Lab, Centre National de la Recherche Scientifique and Grenoble Alpes University, Saint-Martin-d'Hères, France; 2 Speech and Language Laboratory, Centre National de la Recherche Scientifique and Aix-Marseille University, Aix-en-Provence, France; 3 Brain and Language Research Institute, Aix-Marseille University, Aix-en-Provence, France; 4 Anatomy Laboratory, Montpellier University, Montpellier, France; 5 Cognitive Psychology Laboratory, Centre National de la Recherche Scientifique and Aix-Marseille University, Marseille, France; 6 New College, The University of Alabama, Tuscaloosa, Alabama, United States of America; University of Sussex, UNITED KINGDOM

## Abstract

Language is a distinguishing characteristic of our species, and the course of its evolution is one of the hardest problems in science. It has long been generally considered that human speech requires a low larynx, and that the high larynx of nonhuman primates should preclude their producing the vowel systems universally found in human language. Examining the vocalizations through acoustic analyses, tongue anatomy, and modeling of acoustic potential, we found that baboons (*Papio papio*) produce sounds sharing the F1/F2 formant structure of the human [ɨ æ ɑ ɔ u] vowels, and that similarly with humans those vocalic qualities are organized as a system on two acoustic-anatomic axes. This confirms that hominoids can produce contrasting vowel qualities despite a high larynx. It suggests that spoken languages evolved from ancient articulatory skills already present in our last common ancestor with Cercopithecoidea, about 25 MYA.

## Introduction

Language expressed via speech leaves no fossils behind. However, the problem of evolution of the human speech capacity is potentially more easily approached than that of language evolution generally because, while it shares neuro-cognitive mechanisms with language, speech also engages anatomical traits that might leave fossil clues, as well as overt anatomical, physiological, and behavioral aspects for which parallels can be sought in living primates. This study examined potential parallels between human vowels and the vocalic portions of baboon vocalizations.

Grossly, human speech concatenates syllables, each with a vowel at its core and each vowel flanked by consonants. Each language has its own particular phonology (i.e. its own inventory of vowel and consonant phonemes and patterns of their use), but the phonemes are drawn systematically from a universal superset structured by the anatomy and physiology of the vocal tract and vocal folds. In particular, all the vowels are differently situated within a roughly triangular [i a u] vocalic space [1,2]. As a matter of comparative biology, a widespread and longstanding theory [3,4] claims nonhuman primates are incapable of producing systems of vowel-like sounds involving control of their vocal tract, due to their high larynx position and resulting articulatory anatomy. This theory has often been used to buttress the theoretical claim of a recent date for language origin, e.g. 70,000–100,000 years ago [5]. It also diverted scientists’ interests away from articulated sound in nonhuman primates as a potential homolog of human speech, and thus lent support to less direct explanations of language evolution, involving communicative gestures [6], complex cognitive [7] or neural functions [8], or genetics [9].

Several recent discoveries have begun to challenge this dominant view that a low larynx is required for vowel systems. First, descended and dynamically descending larynges have been discovered in animal species with no documented ability to produce systems of vowel-like sounds [10,11]. Second, human infants, with their larynx still high, produce the same range of vowel qualities as adults [12,13]. Third, modeling suggests that the production of vocalic sounds does not depend on the position of the larynx, but rather on the control of tongue muscles and lips to properly constrict the vocal tract [14]. Fourth, simulations also suggest that Neanderthal vocal anatomies supported phonetic capacities equivalent to modern *Homo sapiens* [15]. All these findings reopen the possibility that vocalic systems might very well be present in nonhuman primates, in spite of their high larynx.

Previous studies have already shown certain nonhuman primate vocalizations are vowel-like through acoustic analyses revealing formants [16–22], and also that nonhuman primates can discriminate sounds differing by their formant structures [23]. A pair of studies [24,20] even reported the production of two distinct vowel-like sound in the leopard and eagle calls of Diana monkeys. As noted by Fitch [25], a careful analysis of potential phonetic contrasts by nonhuman primates is desirable in this new context to better illuminate the evolution of human language. The present study has pursued that goal. It combined acoustical analyses of vocalization in baboons with an anatomical study of the baboons’ vocal tract to better examine their capacity to produce and combine vowel-like sounds.

## Methods

### Ethics statement

Animal research conducted at the CNRS is governed by the regulations of the EU and the French Ministère de l′Enseignement Supérieur et de la Recherche. The baboons used for the head and tongue dissections both died from natural causes unrelated to our research project, and before it began. The EU directives and the applicable French rules for ethical treatment of research animals only apply to living animals and do not consider their post-mortem dissections of dead bodies as experiments requiring ethical guidance, so absent any agency to apply to, no approval was available to be requested. However, in accordance with the 2010/63/EU directive on the protection of animals used for scientific purposes, ethical agreement (# 02054.02) was obtained from the CEEA-14 for experimental animal research to conduct audio recordings of the baboons’ vocalizations. Thus, all our research on nonhuman primates was performed in accordance with applicable institutional guidelines of the EU, the CNRS, and the French government.

### Animals and their living conditions

Subjects were 15 guinea baboons (*Papio papio*) living in a larger group of 24 individuals housed at the CNRS primate center, Rousset-sur-Arc, France. The group included males, females, and their offspring, housed in a 25 m X 30 m outdoor enclosure with various climbing structures and connected by tunnels to a 6 m X 4 m indoor enclosure used at night [26]. The baboons were fed daily at 5 pm and water was provided *ad libitum*.

### Audio recording procedure

We recorded and analyzed vocalizations produced spontaneously by the baboons. Recording was carried out from September 2012 to June 2013 between 8:00 am and 21:00 pm, using a*d libitum* techniques, opportunistically sampling social events and responses to stimuli occurring naturally within the baboons’ environment. We particularly focused on the half hour prior to feeding (4:30–5:00 pm), as the baboons were more vocal, and more consistently vocal, during this time. No recording was done from 5 to 6 pm when the baboons were eating, to avoid potential distortion of the vocalizations due to chewing and full cheek pouches. A digital Zoom Handy Recorder H4n (Zoom, Japan: 44.1 kHz sampling frequency, 16-bit resolution, mono) with a Me66 Sennheiser directional microphone (Sennheiser Electronic KG, Germany; with windscreen) was used to record the vocalizations. This is a super cardioid microphone with a high sensitivity (50 mV/Pa ± 2.5dB) and a wide (40Hz– 20 kHz) and flat ± 2.5dB frequency response. Recording was conducted at a distance of < 2 m to 20 m from the baboons, with the greater distances suitable only for long distance vocalizations. Human operators were instructed to avoid social interaction with the subjects and any possible disturbance.

### Corpus

We recorded nearly two thousand spontaneous vocalizations of 3 male (mean age 16 years, range 8–26), and 12 female (mean 13.5 years, range 8–25) social-living adult Guinea baboons (*Papio papio)*. Vocalizations of the young and any adult screams were excluded because their fundamental frequency, sometime approaching 1 kHz, precluded formant detection. Ultimately, from the baboon repertoire, five main types of vocalizations were retained for use, based on presence of observable formants: grunts, wahoos, barks, yaks, and copulation calls. All these vocalizations are well known in the baboon’s repertoire [27]. Grunts are produced by both sexes, copulation calls only by females. Our recordings only garnered wahoos by males and barks and yaks by females, although those calls are sometimes produced by the other sex. Grunts and copulation calls are typically short-distance communications while the wahoos, barks, and yaks carry over longer distances [27].

From our recordings we finally selected a total of 1335 spontaneous vocalizations for analysis, and after splitting the wahoos into their wa—and—hoo phases, the vocalizations we recorded contained a total of 1404 of what we term “vowel like segments” (VLSs): any continuous section within a vocalization containing a consistent and detectable formant structure. These 1404 VLSs served as the corpus for acoustical analyses. All individual segments were isolated, extracted from silence and extraneous noise, and labelled. The upper part of Table 1 reports quantitative data on these segments, including their frequency of occurrence in the database as well as the number of baboons who produced them.

**Table 1:** Recorded corpus characteristics and LPC settings.

	Grunt ♂ & ♀	Wa- ♂	-hoo ♂	Cop ♀	Bark ♀	Yak ♀
**Recorded corpus**						
N baboons	13	3	3	8	11	10
N VLSs	522	69	69	124	116	504
Total duration (s)	65	11	15	10	29	69
Mean VLS duration (ms)	125	159	219	81	250	137
**LPC analysis settings**						
Nb poles	60	30	60	60	30	60
Frame duration (s)	1	1	1	0.5	1	2

Number of baboons producing the VLSs; number of utterances of each VLS; total duration of the file for the VLS (s); mean duration of the VLSs (ms); number of analysis frames for the file; number of poles for the LPC analysis; duration of analysis frames (s).

### General rationale for phonetic analyses

To adapt well-known human speech science techniques to the search for vocalic elements in non-human primates, the basic outline of our methodology was as follows:

- apply Linear Predictive Coding (LPC) analysis [28,29] to all selected VLSs, to extract the first two formants (F1 & F2), and use autocorrelation to determine the fundamental frequency (F0).
- locate these VLSs in an appropriate F1-F2 space, the Maximal Acoustic Space (MAS) normalized for the baboon vocal tract
- label these VLSs with transcription symbols from the International Phonetic Alphabet (IPA) [1], standard in human phonetics [30,31].
- use these IPA phonetic labels to determine the corresponding articulatory shapes, well known in human speech, and, through an anatomical study of tongue musculature, confirm the baboons’ ability to produce these shapes.

**Fig 1:**
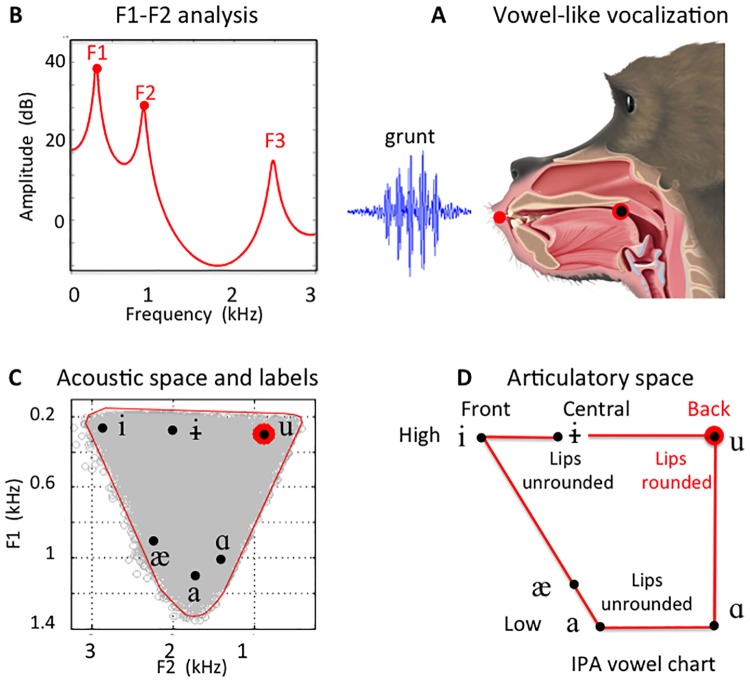
Procedure for acoustic analysis and VLS labeling. (A) Vocalizations in both human and nonhuman primates use the acoustic signal from the vocal folds vibrating at their fundamental frequency (F0). The formant frequencies depend on the configuration of the vocal tract and the lip opening. (B) LPC analysis was used to reveal the formants of each VLS (supplemental information S2 Fig) [28,29]. (C) A Monte Carlo procedure using an n-tube model normalized for the anatomical measures of the baboons’ vocal tracts then served to generate the MAS (shown by the red line). With this normalized MAS reference, any VLSs could be precisely labeled with the IPA vowel symbols [30,31]. (D) The VLSs thus labeled correspond to well-documented articulatory configurations with characteristic tongue positions and lip openings. (A-D) Red-&-black dots indicate the corresponding values for this illustrative grunt vocalization, which is classified as [u].

Fig 1 schematically represents this procedure using graphics pertinent to each step. See the Supplement for expanded discussion of these four basic aspects of this study.

#### LPC analyses of the VLSs

For acoustical analyses, the VLSs were grouped into one file per vocalization type (e.g., bark), except for the two phases of the wahoos (wa- and -hoo), which were split and grouped into separate files. To retrieve formants from each file, LPC [28,29] and peak detection analysis was carried out, after pre-emphasis by derivation. Like many acoustic techniques, LPC works best on long signals recorded cleanly in laboratory conditions, whereas our VLSs are short and were recorded outdoors in varying conditions. To limit the perturbation due to noise and to maximize the fidelity of the LPC results and achieve the clearest possible characterization of our VLSs, our acoustic analysis was performed using frames from 0.5 to 2 seconds long, so that each frame encompassed several utterances. Analysis was done with successive frames operating as a sliding window overlapping by half each step, and our results and subsequent processing are based on the frame outputs from this LPC processing. The frame database was then filtered to further control for detection errors, and all the frames missing F1 or with F1 or F2 values greater than 3 standard deviations from the means of their VLS categories were eliminated from the dataset (see below). Also, F0 was measured in the same frames using autocorrelation and peak-picking.

There is no theoretically definitive method for setting the number of poles in LPC formant detection, so they must be set empirically [32], considering sampling rate, frequency range analyzed, and especially fundamental frequency of the signal. As indicated in the bottom part of Table 1, we chose settings of 30 poles for high F0 VLS (male “wa-” and female “bark”), and settings of 60 poles for the low F0 VLSs (all other VLSs). The supplement provides expanded discussion with illustrative examples of the intricacies of LPC behavior relating to F0 that led us to the settings we have used (supplemental information S1 and S2 Figs). Note also that the number of poles we have selected is consistent with previously published works. Menard et al. [33] for instance used 10–14 poles at 22.050 kHz sampling for high F0 children’s voices and Owren et al. [17] used 14 poles at 10 kHz sampling for low F0 baboon vocalizations. Extrapolating to our higher sampling rate of 44.1 kHz, our chosen settings of 30 poles for high F0 and 60 poles for low are entirely comparable to the settings in those studies. To further test the validity of those settings, we ran analyses dividing both sampling rate and poles in half (respectively 15 and 30 poles), and a t-test showed the differences in mean formant frequencies (2.6 Hz for F1, 9.6 Hz for F2) to be non-significant (p = 0.265 and p = 0.197, respectively). This analysis further confirms that our formant measurements are robust across a range of LPC settings.

#### Computation of the maximal acoustic space (MAS)

When acoustically excited, a fixed tube of any given configuration can only produce a single fixed pattern of resonance. However, the vocal tract is mobile, not fixed, across vocalizations, with length varying somewhat, with cross-sectional areas varying by more than an order of magnitude, and with its constrictions and cavities shifting along its length, so it can produce an inventory of different resonance patterns. Appropriately sampling the attainable physical configurations of such a tube allows us to determine its maximal acoustic space (MAS), which is defined as the possible formant configurations generated by all possible physical configuration of the tube. A MAS can be represented by the multidimensional acoustic space determined by the number of formants considered, typically 2D for an F1 x F2 space. By definition, any signal filtered by a tube (or vocal tract) of a given length will have its first two formants within the F1-F2 MAS, regardless of the tube shape. We have previously shown [14] that the MAS can be calculated for a tube of any given overall length, using the well-known technique of subdividing this tube into *n* adjacent cylindrical components [34,35] and varying their lengths and cross-sectional areas through an appropriate range, while maintaining the overall length. (See also the supplement for conceptual background and development of the MAS.) We have also shown that the 2D (F1-F2) MAS is adequately approximated when the number of tube sections is at least 4 [14]. The effect of any vocal tract curvature has been shown to be negligible [36], so it is typically modeled as straight. Knowing the length of a given vocal tract, it is thus possible to calculate its MAS, regardless of any anatomical peculiarities. The MASs for the male and female baboons were computed from a total of 100,000 Monte Carlo simulations, where number of tubes n = 4 and the cross-sectional area of each tube was selected randomly from 13 possible values logarithmically distributed between Amin = 0.125 cm^**2**^ and Amax = 8 cm^**2**^. In our study, the total tube length was set to 13.5 cm for the males and 11 cm for the females, to agree with anatomical data obtained from dissections (see below).

#### Phonetic labeling and inferences on articulatory gestures

As with humans, the calculated MAS served as a vowel space in which to situate the formant measurements for baboon vocalizations. Then the phonetic labels of the VLSs were identified by comparison to previously labeled human data, in our case the MAS and the vowels of American English children [30,31] with an estimated vocal tract length of 12 cm [37] (about the same length as measured in our dissections, described below, of the baboons' vocal tract). It is one of the fundamental tenets of the IPA that each phonetic symbol is associated with a particular configuration of the tongue and lips (cf. Fig 1D). Our final question, addressed below in the anatomical part or our study, is whether such configurations are articulatorily possible for baboons.

### Tongue dissection

The heads of one male and one female adult Guinea baboon were dissected to measure their vocal tract and vocal fold lengths, and examine the tongue muscles in details (supplemental information S3 Fig). This anatomical study was conducted on two baboons obtained from CNRS-UPS 846 biobank, after their deaths by natural causes. The vocal tract lengths (13.5 cm for the male and 11 cm for the female) approximate human vocal tract lengths typical of a 12.5-year-old boy and a 8-year-old girl, respectively [38]. The baboons’ vocal folds measured 16.5 mm for the male and 11 mm for the female, in the same range as those of adult humans [39]. Thus, compared with humans, baboons have a child-like vocal tract but adult-like vocal folds. This discrepancy affects our perception of their VLSs, and disrupts auditory phonetic labeling, thus necessitating the MAS procedure described above.

## Results

### Acoustical analyses

**Fig 2:**
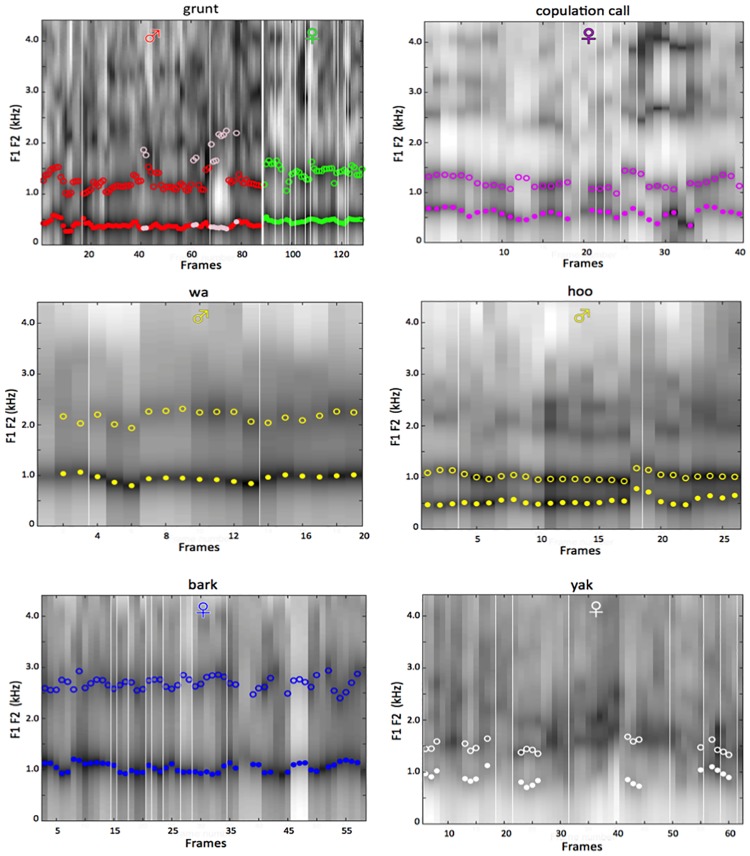
LPC spectrograms and formants, by VLS class. The panels show LPC spectra for all frames. The white bars approximate the boundaries between sexes (thick bar, Grunt panel) and individual animals (although given our sliding window procedure with frames overlapping, the actual boundary is typically internal to the frames both preceding and following each bar). Frames were selected for further use when both F1 and F2 were detected by LPC (within plausible ranges) and were within ± 3 standard deviations of their class means; those frames are indicted by a dot for F1 and an open circle for F2 at their measured frequencies in those frames. The acoustic results reported were calculated from the frames thus selected. See the supplemental section for additional details on these LPC analyses.

**Fig 3:**
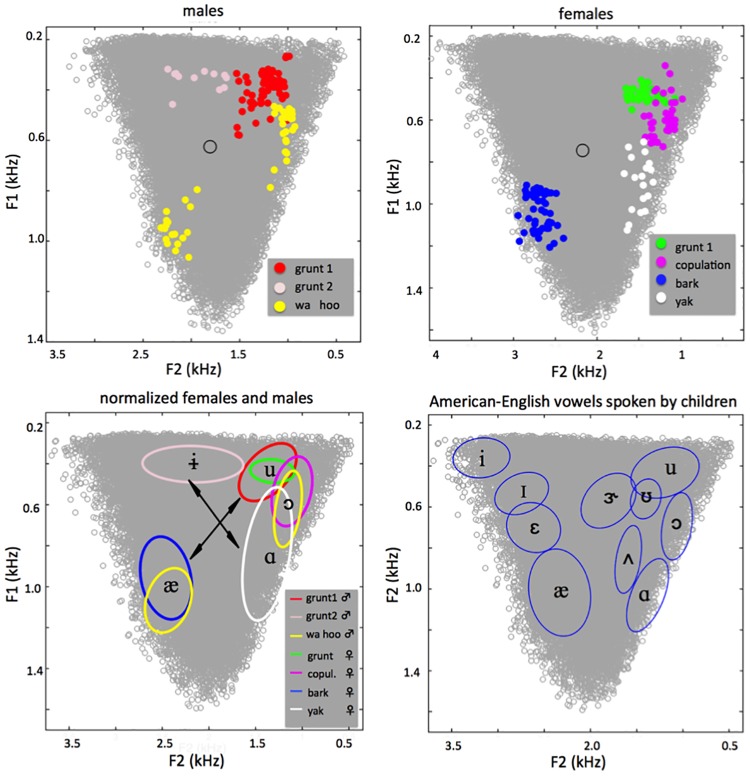
Distribution of VLSs within the MAS. (A) and (B) show the males’ and females’ MAS, respectively, with our data (analyzed in frames). An open circle marks the location where a neutral tube of the vocal tract’s length would produce the central schwa sound, [ə]. (A) confirms that male grunts occur in two subtypes, grunt 1 and grunt 2, based on distinct F2 ranges. (C) shows normalized data, pooling males and females. Ellipses within the MAS delineate an area covering 86.5% of the data for each VLS category. Note that the baboons produced five distinct VLSs, [ɨ æ ɑ ɔ u]. Comparison of the findings to those of American-English speaking children [30, data publicly available in Praat] shown in (D) demonstrates strong similarities between the two species, suggesting a phylogenetically ancient origin of the vowel systems of humans. Arrows indicate acoustic axes.

The acoustic analyses described above render results that we now present in three different forms. First, Fig 2 gives a spectrographic representation of the frame-by-frame LPC results for all analyzed frames, before filtering out frames with detection errors. This figure confirms the presence of formants in all VLSs, and shows that those formants are grossly similar within class and different across classes. Two special cases must be noted in these spectrograms, and also in Table 2 and Fig 3, following: Because of separate F2 distributions, the grunts for males actually exist in two different forms, which we term grunt 1 (shared with females) and grunt 2. This is discussed further in the Supplement. Note also that the high frequency and the periodicity characteristics of voicing in yaks render measurement of F1 and F2 problematic for a large number of yak frames. This issue is also discussed in the supplement. Table 2 then reports summary statistics for the frames retained (i.e., with good formant detection), specifically the F0, F1 and F2 means and standard deviations for each VLS class. Finally, Fig 3 shows both frame data and enclosing ellipses for the VLSs in the MAS’s F1 F2 acoustic space, and how that compares to vowels for human 12-year-olds, with their comparable vocal tract length.

**Table 2:** Corpus frame statistics and acoustic results.

	Grunt 1 ♀	Grunt 1 ♂	Grunt 2 ♂	Wa- ♂	-hoo ♂	Cop ♀	Bark ♀	Yak ♀
**Corpus frame statistics**								
Total nb of frames	40	77	13	20	26	39	56	62
Nb of frames selected	39	76	12	18	26	36	50	19
Nb (%) frames eliminated	1 (3)	1 (2)	1 (8)	2 (10)	0 (0)	3 (8)	6 (11)	42 (68)
**Acoustic Results**								
F1 (Hz)	476	392	357	948	552	583	1044	916
	(31)	(63)	(40)	(70)	(82)	(93)	(89)	(140)
F2 (Hz)	1440	1219	1932	2165	1025	1211	2685	1500
	(129)	(137)	(240)	(112)	(66)	(119)	(121)	(116)
F0 (Hz)	64	61	71	417	121	133	431	—
	(20)	(20)	(25)	(105)	(37)	(56)	(45)	—

Total number of frames; number of frames selected, number and percentage of frames eliminated, mean and standard deviation (for selected frames) for the first two formants and for the fundamental frequency (after autocorrelation).

This evidence from our acoustical analyses reveals that baboons produce at least five distinct classes of VLSs, each requiring a different tongue position in the vocal tract. These five VLSs correspond to the high central [ɨ], high back [u], mid-high back [ɔ] low front [æ] and low back [ɑ]. None of these VLSs is located where, in baboons as in humans, a neutral tube of the appropriate length would produce the central schwa [ə] (at the cross in Fig 3A and 3B). Thus, these findings of five distinct VLS classes constitute five separate counterexamples to the claim that nonhuman primates are restricted to schwa-like productions [4]. Moreover, VLS locations, along the edges of the MAS, reveal contrasts along 2 axes (Fig 3) comparable to the vertical and horizontal tongue movement dimensions which are universal in human speech and are therefore the organizing principle of the IPA vowel chart (Fig 1D). Along the [æ] ⇔ [u ɔ] axis we find the males’ bark and wa- as [æ] opposed to the males’ grunt 1 and -hoo and the females’ grunt 1 and copulation call as [u ɔ]. The second [ɨ] ⇔[ɑ] axis opposes the [ɨ] from the males’ grunt 2 and the [ɑ] of the females’ yak. This recognition of multiple VLSs in the baboon inventory makes two further observations indispensible, since they make revealing points about the relations among those different VLSs. First, we found that the [ɔ] quality occurs both in the copulation calls produced only by females and in the—hoo of the wahoo produced mainly by males. Likewise, [u] occurs in both the grunt 1 of females and the grunt 1 of males, and [æ] in the bark of females and the wa—of males. Thus, three instances show that a single VLS can be used in two different vocalizations by two different classes of individuals. Second, data further reveals that baboons regularly produce two distinct VLSs consistently and in succession within a single utterance, specifically, the [æ] and the [ɔ] in the wahoo.

**Fig 4:**
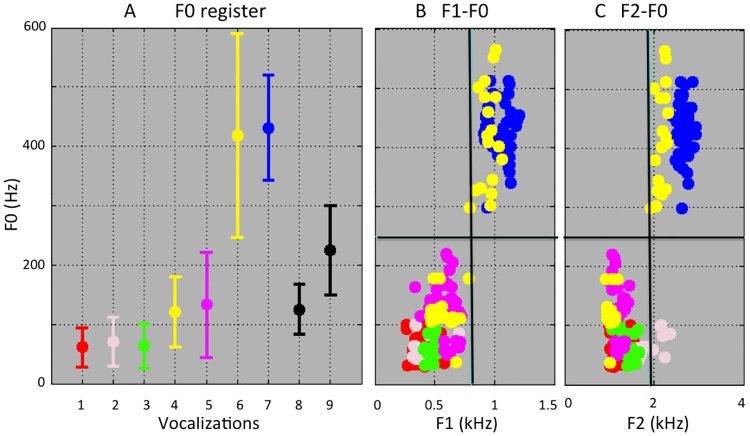
Fundamental frequency in baboon VLSs. (A) Baboon F0 by VLS and sex (mean and two SDs). For comparison, black bars show typical F0 for conversational speech by human men and women [37]. (B, C) For most VLSs, F0, F1, & F2 were either all high in their ranges (6 wa- ♂, 7 bark ♀) or all low (1 grunt1♂, 3 grunt ♀, 4 -hoo ♂, 5 copulation ♀), although grunt2 ♂ was characterized by a low F0 and high F2 (C).

We also found that the VLSs’ F0 frequencies varied (see Fig 4A) from 30 Hz (grunts) to 600 Hz (wa-), a range that constitutes, at this location in the frequency scale, approximately four and a half octaves. By comparison, F0 ranges across about one octave in human conversational speech. Differences in F0 were observed between the two sex’s VLSs, and between the wa- and -hoo segments (Fig 3A). VLS scatterplots on F0 and F1 (Fig 4B), and on F0 and F2 (Fig 4C), categorically separate the two VLS groups that define the [æ] ⇔ [u ɔ] axis mentioned above. These findings demonstrate partial coupling of F0 (produced by the vocal folds) and of the first two formants (produced by the vocal tract). This contrasts sharply with human speech production, where F0 (intonation) and F1-F2 (vowels) are controlled independently.

### Tongue anatomy

**Fig 5:**
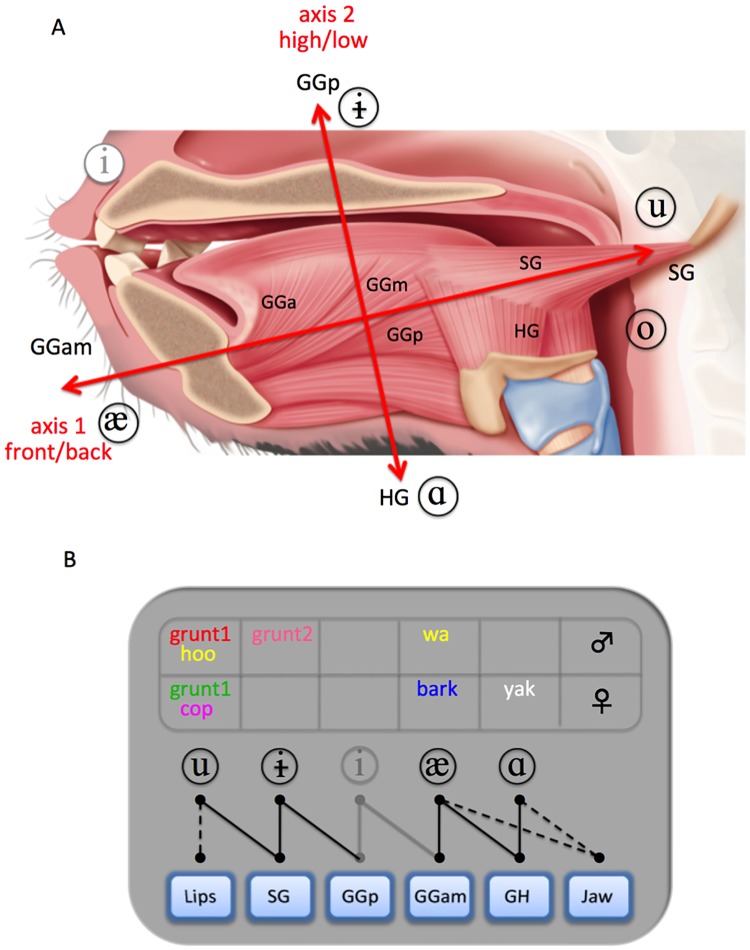
Anatomical structure of the baboon tongue and muscle recruitment during VLS production. (A) The baboon’s muscle fiber orientation allows tongue motion along two main axes (see also supplemental information S3 Fig). The first axis produces the front/back contrast [æ] ⇔ [u ɔ], including the [u] VLS, which requires a constriction in the back of the vocal tract. Movement along this axis uses antagonistic activation of GGam and SG tongue muscles. The second axis produces the [ɑ] ⇔ [ɨ] VLS contrasts by controlling vertical tongue displacement using the GGp and HG tongue muscles. (B) The baboons’ different VLSs can each be explained by recruitment of a unique configuration of tongue muscles. GGa, GGm, GGp: anterior, medium, posterior part of the genioglossus; HG: hyoglossus; SG: styloglossus.

Our anatomic study documented important similarities between human and baboon tongue musculature. Although longer, the baboon tongue has the same muscles as a human tongue (see Fig 5 and supplemental information S3 Fig), with a shape and proportions similar to a child’s tongue. The combined evidence from this dissection, EMG studies [40,41], and biomechanical models of humans [42,43] implies that baboons have all the articulatory effectors required both to produce the formant structure of their documented VLSs (Fig 5A), and to move their tongues along the two axes we have discovered (Fig 5B). This species can therefore produce its distinct VLSs despite a high larynx, in sharp contradiction with Lieberman’s hypothesis [4,3].

## Discussion

The main findings of our study can be summarized as follows: First, our study confirms that baboon vocalizations contain different kinds of VLSs, and that these VLSs have certain consistent traits. These include distinctive formant patterns that justify grouping them into the five classes of VLS we have found, each of which is comparable to a human vowel as charted by the IPA. Second, we document that baboons produce two distinct VLSs consistently and in succession within a single vocalization, specifically, the [æ] and the [ɔ] in the wahoo. Third, the [ɔ] quality occurs in the copulation calls produced only by females and in the—hoo of the wahoo produced mainly by males, so a single VLS can be used in different vocalizations, comparably to different phonemes in human languages. Finally, our study shows that the five VLSs documented involve two acoustic axes produced by motion of the tongue in horizontal and vertical axes, in a manner clearly comparable to the two articulatory-acoustic dimensions universal to human speech. Taken together, these findings demonstrate that the baboons have a much richer system of VLSs than previously documented, in spite of their high larynx.

Human vocal communication uses a phonological system wherein words are distinguished by the contrasts among their constituent phonemes (grossly, vowels and consonants). These are drawn from an inventory that has been documented in different languages as ranging from 11 to 141 phonemes, including from 3 to 24 vowels [2]. As an example, in American English, phonology distinguishes the words *boat* (/bot/) and *bat* (/bæt/) exclusively through the distinction between the /o/ and /æ/ vowel phonemes they contain. Here we report for the first time that the vocal repertoire of a single nonhuman primate species contains at least a set of five distinct VLS, some found in the vocal productions of the males or females only, and others in both sexes.

Our findings therefore reveal a loose parallel between human vowels and baboon VLSs by demonstrating that both have a phonetic inventory of vocalic qualities differentiated by formant structure and that these structures are characteristic properties of vocalizations produced in distinct social contexts or for different functions. From an evolutionary standpoint, demonstration of a two-axis vocalic proto-system in baboons suggests that the human vocalic system did not emerge *de novo* but originates from articulatory capacities already present in our common ancestors. We believe that the currently dominant view, that vowel systems can only have emerged after the descent of the larynx in modern *Homo sapiens*, is falsified by our finding of 5 distinct vowel qualities in a 2 dimensional system in an old world monkey, the Guinea baboon (*Papio papio)*.

In human languages, formants vary independently from the laryngeal frequency, and this not what we found in baboons. This aspect of our findings has implications for our understanding of language evolution. F0 and formants were apparently entangled (Fig 4) during speech evolution’s early stages, although [ɨ] (from grunt 2) seemingly escapes this link between F0 and F2 and might reflect an early dissociation between F0 and formants. Clearer dissociations between F0 and the formants must have emerged later in the hominin lineage, probably accompanied by more complete coverage of the vowel space. We suggest that vowel quality differences were progressively more exploited for human communication, with evolution of increasingly precise shaping of the vocal tract in the hominid line. These vowel quality differences eventually developed into the phonological systems using contrasts based on species-wide mastery of the articulatory dimensions universal in modern humans and documented in the International Phonetic Alphabet.

Whatever the course of the emergence of language and speech, the evidence developed in this study does not support the hypothesis of the recent, sudden, and simultaneous appearance of language and speech in modern *Homo sapiens*. Rather, our findings in a monkey species allow us to infer certain features of ancestral communication systems antedating our own species. Specifically, since we show that baboon VLSs use 5 distinct vocalic qualities organized in an articulatory-acoustic system similar to that of humans, we conclude that a homologous proto-vocalic system must now be inferred in our last common ancestor with Cercopithecoidea, about 25 MYA, and that that system was a precursor to the vowel systems universal in spoken human language.

## Supporting Information

S1 Fig Using pole settings to avoid LPC formant detection errors. Example LPC analyses of two grunts (top) and two barks (bottom), with 30 poles (red) and 60 poles (blue) superimposed on an FFT analysis. Both LPC & FFT calculated using MATLAB. For the grunts (F0 low) only the LPC with 60 poles fits the FFT well. LPC with 30 poles misses the first formant in the left grunt and the second formant in the grunt on the right. On the other hand, for the barks (F0 high) the FFT is well fitted with 30 poles and the formants are well detected. With 60 poles, spurious peaks related to harmonics are erroneously detected.
(TIF)

S2 Fig Spectrograms. Examples of spectrograms (from Praat, available at http://www.fon.hum.uva.nl/praat/) and overlaid FFT and LPC spectra (calculated using MATLAB) for grunts (♀♂), copulations calls (♀), wa- (♂), -hoo(♂), barks (♀), yaks (♀). (LPC was set to 60 poles for grunts, copulations calls (♀),-hoo(♀) and yaks, 30 poles for barks, and wa-. Sampling frequency was 44.1 kHz.
(TIF)

S3 Fig Anatomy of the tongue. Anatomic sagittal view of the head of a female baboon: (1) hyoid bone, (2) air sac, (3) thyroid cartilage, (4) epiglottis, (5) arytenoid cartilage, (6) vocal folds and glottis, (7) cricoid cartilage, (8) trachea, (9) lips, (10) incisors, (11) mandible, (12) hard palate, (13) velum, (14) pharyngeal wall, (15-16-17) anterior GGa, medial GGm, and posterior genioglossus GGp,(18) superior longitudinalis, (19) geniohyoid GH, (20) digastric anterior, (21) C1, (22) C2,(23) C3, (24) mid sagittal line of the vocal tract used to infer the tract length and the computation of the MAS. Note the orientation of the fibers of the GGa, GGm and GGp muscles, which approach vertical on the anterior part of the tongue but are effectively horizontal in the posterior part. The fibers of the styloglossus (SG) muscle on the lateral sides of the tongue have approximately the same inclination as those of a human baby [10]. As in humans, the hyoglossus (HG) muscle has two components which are inserted into the body of the hyoid bone and over the entire extent of the great horn. Its fibers are oriented vertically as found in human children. (N.B.: SG and HG are both lateral to the midline, and do not appear on this view.) This anatomical study shows that a baboon’s tongue has the same musculature as a human’s. Regarding shape and proportions, the baboon’s tongue is more similar to that of a child than that of a human adult.
(TIF)

S1 File Supporting information. Complementary information on the rationale of the method, parameter settings for LPC analyses, MAS computation and normalization, results, and data file and software accessibility.
(DOCX)
