# Supplementary figures and images for: Evidence of a Vocalic Proto-System in the Baboon (Papio papio) Suggests Pre-Hominin Speech Precursors
Source: PLoS One. 2017 Jan 11;12(1):e0169321. doi: 10.1371/journal.pone.0169321 (PMC5226677; doi:10.1371/journal.pone.0169321)

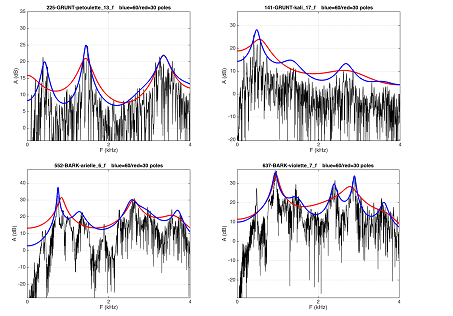

Supplement: S1 Fig — Example LPC analyses of two grunts (top) and two barks (bottom), with 30 poles (red) and 60 poles (blue) superimposed on an FFT analysis. Both LPC & FFT calculated using MATLAB. For the grunts (F0 low) only the LPC with 60 poles fits the FFT well. LPC with 30 poles misses the first formant in the left grunt and the second formant in the grunt on the right. On the other hand, for the barks (F0 high) the FFT is well fitted with 30 poles and the formants are well detected. With 60 poles, spurious peaks related to harmonics are erroneously detected. (TIF) [file pone.0169321.s001.tif]

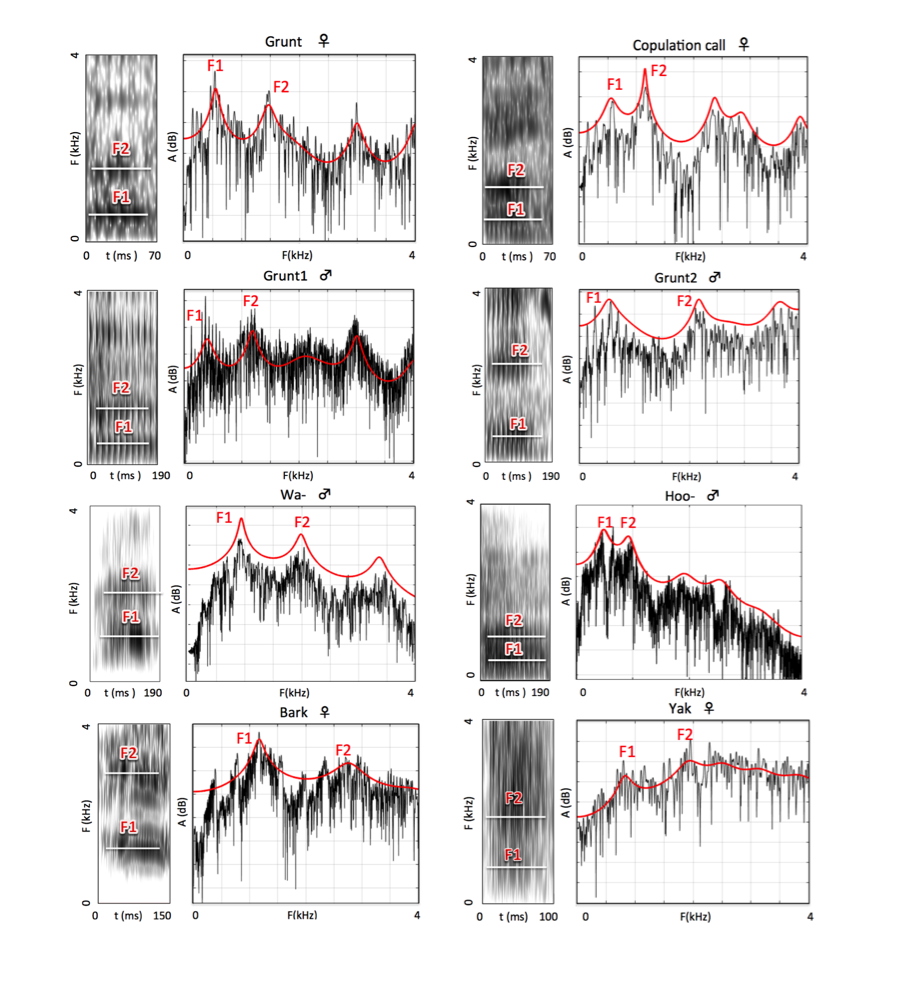

Supplement: S2 Fig — Examples of spectrograms (from Praat, available at http://www.fon.hum.uva.nl/praat/) and overlaid FFT and LPC spectra (calculated using MATLAB) for grunts (♀♂), copulations calls (♀), wa- (♂), -hoo(♂), barks (♀), yaks (♀). (LPC was set to 60 poles for grunts, copulations calls (♀),-hoo(♀) and yaks, 30 poles for barks, and wa-. Sampling frequency was 44.1 kHz. (TIF) [file pone.0169321.s002.tif]

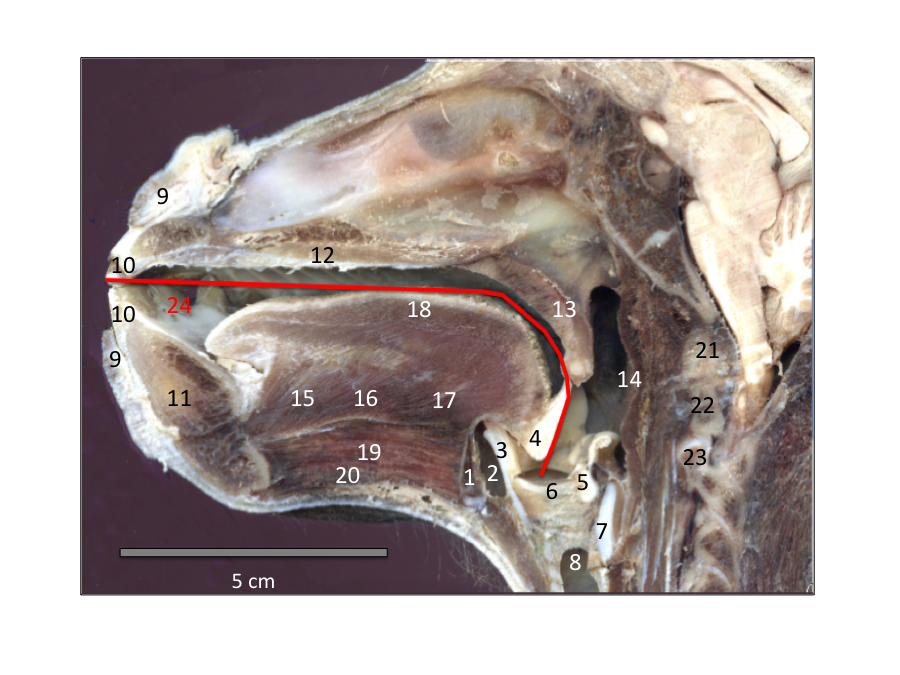

Supplement: S3 Fig — Anatomic sagittal view of the head of a female baboon: (1) hyoid bone, (2) air sac, (3) thyroid cartilage, (4) epiglottis, (5) arytenoid cartilage, (6) vocal folds and glottis, (7) cricoid cartilage, (8) trachea, (9) lips, (10) incisors, (11) mandible, (12) hard palate, (13) velum, (14) pharyngeal wall, (15-16-17) anterior GGa, medial GGm, and posterior genioglossus GGp,(18) superior longitudinalis, (19) geniohyoid GH, (20) digastric anterior, (21) C1, (22) C2,(23) C3, (24) mid sagittal line of the vocal tract used to infer the tract length and the computation of the MAS. Note the orientation of the fibers of the GGa, GGm and GGp muscles, which approach vertical on the anterior part of the tongue but are effectively horizontal in the posterior part. The fibers of the styloglossus (SG) muscle on the lateral sides of the tongue have approximately the same inclination as those of a human baby [10]. As in humans, the hyoglossus (HG) muscle has two components which are inserted into the body of the hyoid bone and over the entire extent of the great horn. Its fibers are oriented vertically as found in human children. (N.B.: SG and HG are both lateral to the midline, and do not appear on this view.) This anatomical study shows that a baboon’s tongue has the same musculature as a human’s. Regarding shape and proportions, the baboon’s tongue is more similar to that of a child than that of a human adult. (TIF) [file pone.0169321.s003.tif]
