# Supplementary material for: Evidence of a Vocalic Proto-System in the Baboon (Papio papio) Suggests Pre-Hominin Speech Precursors
Source: PLoS One. 2017 Jan 11;12(1):e0169321. doi: 10.1371/journal.pone.0169321 (PMC5226677; doi:10.1371/journal.pone.0169321)
Supplement: S1 File — Complementary information on the rationale of the method, parameter settings for LPC analyses, MAS computation and normalization, results, and data file and software accessibility. (DOCX) [file pone.0169321.s004.docx]

## Supplemental Text

# Methods

## Rationale

We elaborate below on our four-point rationale in more detail. This overall procedure we have adopted for phonetic labeling and then articulatory interpretation of those labels is also illustrated in Fig 1.

(i) The Source-Filter theory [1,2] sets out the acoustic principles whereby articulation results in transmitted sound as the acoustic source signal, generated by vocal fold vibration, is modified by the resonance patterns implicit in the cavity configuration of the vocal tract. Those modifications result in amplified regions in the acoustic spectrum, which are known as formants. Formants are inherent to any type of vocal production since the vocal tract will always have resonant properties and thus always produce formants. Human vowels are characterized by relatively long steady state formant patterns determining their identity, unlike consonants, which are characterized in part by formant transitions at their boundaries with the contiguous vowels. Formant configurations, in steady-states and transitions, thereby convey information about vocal tract shape, whether static or dynamic. In our study, we used LPC analysis, explained in more detail below, to extract the formant values of our recorded VLSs for use as described as follows:

(ii) Formants extracted by LPC analysis must be set in an acoustic vowel triangle that serves as a reference space for their identification in terms of vocalic class. To achieve this, it is necessary to get the correct reference space in two steps, first a step of vowel normalization, then the construction of an appropriate vowel space, the MAS, both of which we describe below in the “MAS theoretical background, computation and normalization” section. Any phonetic labeling of the baboon VLSs involving human auditory judgment would be suspect due to our fundamental frequency (F0) range differences. Thanks to the normalization process, this difficulty is alleviated, and direct human/baboon comparisons using IPA symbols is possible. This process is objective, and independent of human perception of the VLS quality.

It bears noting that Lieberman [3] has raised criticisms of the Variable Linear Articulatory Model (VLAM, the advanced articulatory model of the vocal tract we have developed and used in previous studies), and that we have replied [4], but those criticisms would not apply here since we have not used VLAM in this study. Here, we have generated the MAS with a 4-tube model, relying solely on the general acoustic properties of tubes.

(iii) The articulatory universals of the production of vowels include the horizontal position of the tongue, the vertical position of the tongue and jaw, as well as the shape (round vs. spread) and aperture of the lips. These three universal dimensions are the organizing principles of the vowel chart of the International Phonetic Alphabet [5]. The [u] vowel illustrates the link between formants and tongue position, as it is produced by a human tongue at its highest and most rearward position, together with lip rounding and a small lip opening, resulting in a low F1 (first formant, counting from low to high) and a low F2. When a comparable low F1/ low F2 configuration is obtained in any vocal signal (relative to the appropriate acoustic space), it is therefore possible to label this production as [u] given the well-established correspondence between vowels formants and the vowel articulations defined by the IPA. Based on the systematic relationship between the F1/F2 configuration and the position of the human tongue, the converse inference is also possible: to infer the horizontal (back) and vertical (high) position of the tongue and the shape of the lips (rounding) when this specific configuration of low F1/ low F2 formants is produced. Our study used this general principle to label the baboon’s vocalization and infer their analogous tongue position.

(iv) Finally, in light of the articulations implied by the phonetic labeling, our anatomical study aims to show that the subset of the tongue positions associated with the baboon VLS formants are achievable by their muscular equipment, and then to show that the structure of this subset constitutes a proto-system. From an evolutionary point of view, this subset could then serve as a basis for studying the phylogeny of speech.

### Parameter settings for LPC analyses

Linear predictive coding (LPC) is an audio analysis technique for determining the spectral envelope of a signal derived from the filter effects of a tube on a periodic source. In speech analysis it is commonly used to locate formants in vocalic utterances, as we are doing here for vocalic portions of baboon utterances. There is (as stated in the main text) no theoretically definitive method for setting the number of poles in LPC formant detection, and they must be empirically adjusted due to two known types of formant detection error that LPC is prone to. On one hand, when the spectrum is densely activated by the closely-spaced harmonics from a low fundamental frequency (F0), LPC analysis with too few poles may poorly track the relatively rich spectral information, and as a result miss some of the formants altogether. On the other hand, when the spectrum is sparsely activated by the more widely-spaced harmonics from a high F0, LPC with too many poles may falsely designate separate harmonics as formants, rather than the broader-band formant they incompletely activate. We can demonstrate both such potential errors with our own data.

The two upper panels of Fig S1 illustrate two of our recorded vocalizations, specifically two grunts, which are a low-F0 [u]-like VLS with an F1 around 400 Hz and an F2 around 1500 Hz. The figure shows LPC analyses with two different pole settings overlaid on an FFT (Fast Fourier Transform) base representation. The LPC in red uses 30 poles, while the LPC in blue uses 60. As we clearly see, the red LPC with 30 poles misses F1 in the left panel and F2 in the right panel. The blue LPC with 60 poles correctly detects F1, F2, and also F3 in both panels.

By contrast, the two lower panels of Fig S1 illustrate two barks, which are high F0 [æ]-type VLSs with F1 around 1000 Hz and F2 around 2700 Hz. As before, the red LPC uses 30 poles and the blue 60, and as we see, the blue line has a spurious peak between the first two formants in the left panel and two spurious peaks in the right panel, all of which are due to misidentifying harmonics as formants.

The number of poles we have set for our LPC analyses (see Table 1) alleviates these issues. As indicated in the main text (see references there), they are also consistent with previously published works.

### MAS and normalization

Sexual dimorphism in primates generally and in baboons in particular led us to expect vocal tract length differences here as well, and our dissection confirmed our expectation, with the vocal tract of the male measuring 13.5 cm and of the female, 11 cm. Thus, for baboons (male and female) as for humans, this difference must be taken into account in generating MASs with the 4-tube model, for use in identifying vocalic qualities in the resulting formant structures. The anatomical parameters retained for our use of the N-tube model are provided in the main text.

Suitable normalizing transforms allow objective comparisons across sexes and, in humans, across. In our study, the MAS is generated for a given length Li and then it is uniformly transformed in a MAS for length Lj using the equation:

F_n,j_ = F_n,i *_ L_i_/L_j_ .

Here Fn represents any of the numbered formants, L represents the length of a vocal tract, and i and j index two vocal tracts of different lengths. Given a formant frequency Fn,i produced by vocal tract or a model with known length Li, this equation gives the frequency of the same Fn formant as it would be produced by a vocal tract with the same cross-sectional areas but with all the corresponding tubes proportionally shifted to a different overall length.

We have used the same normalization equation to bring the formant values of recorded VLSs of male and female baboons, with vocal tract lengths of 13.5 cm and 11 cm respectively, into an MAS designed to represent the vowel space of the Peterson & Barney child subjects, who have been estimated to have a vocal tract length of 12 cm [6] corresponding to an age of approximately 12 years [7]. Since this is between the measured lengths of the two individual baboons we examined, the resulting MAS is an appropriate base from which to identify the phonetic classes of the normalized VLSs.

# Results

### Acoustical analyses

The specifics of the findings about grunts and yaks merit discussion. Examination of the F2 of the grunts produced by the males showed a bimodal distribution. Most of the males’ frames, like all of the females’, had F2 distributed from 1 to 1.55 kHz, while another smaller number had F2 ranging from 1.65 to 2.2 kHz. The majority of grunt frames were in the [u] area of the MAS, while the MAS location of the smaller group of frames makes it clear that this is a second, separate VLS, corresponding to a different, more fronted IPA transcription symbol, [ɨ]. The more common grunts we have termed grunt 1, and those with the higher F2 we term grunt 2. Grunt 1 corresponds to the grunt vocalizations described by Rendall et al., for instance [8]. The behavioral function of grunt 2 remains unknown at this point, but they may correspond to the "high frequency grunting" described by Hall & DeVore [9] as produced by males when they are close to and possibly about to touch an infant. We worked out descriptive statistics for the two grunts separately, as reported in Table 2.

Yaks have an exceptionally high fundamental, sometimes above 1 kHz, and often with irregular periodicity, making its measurement inexact. When F0 is above the F1, the formant is not acoustically activated, and even if low enough, irregularities in voicing can reduce its amplitude. As a result, only about half the frames have a formant around 0.9 kHz, while all the frames have a formant around 1.5 kHz. Those with both occupy an area in the MAS that corresponds to the [ɑ] IPA symbol. If the others had their F1 around 1.5 kHz and their F2 ranging from 2.0 to 3.0 kHz, as seems likely from the spectra in Fig S2 then the yaks would occupy two entirely different areas in the vocalic space, and there is no prior auditory or articulatory observation, by us or in the literature, to support such a claim. Moreover, the second distribution would lie in large measure outside the MAS, which is acoustically impossible. We must thus infer that the LPC in these cases failed to detect the F1 for those yak frames with no formant around 0.9 kHz. Because of the problem detecting F1, we chose to eliminate all the affected frames despite the high loss rate, and calculate the descriptive statistics only on the basis of the 19 selected frames with both F1 and F2 detected, thus treating yaks according to the same criteria as the other VLS classes. The results are reported in Table 2.

# Datafile and software accessibility

Sound datafiles are available at:  [http://doi.org/10.17605/OSF.IO/3NDFH](http://doi.org/10.17605/OSF.IO/3NDFH)

Software for LPC analyses are available at: [http://www.gipsa-lab.grenoble-inp.fr/valorisation/logiciels.php?id_valorisation=135](http://www.gipsa-lab.grenoble-inp.fr/valorisation/logiciels.php?id_valorisation=135)

Software for MAS computation is available at: [http://www.gipsa-lab.grenoble-inp.fr/valorisation/logiciels.php?id_valorisation=134](http://www.gipsa-lab.grenoble-inp.fr/valorisation/logiciels.php?id_valorisation=134)

**Supplemental Figures**


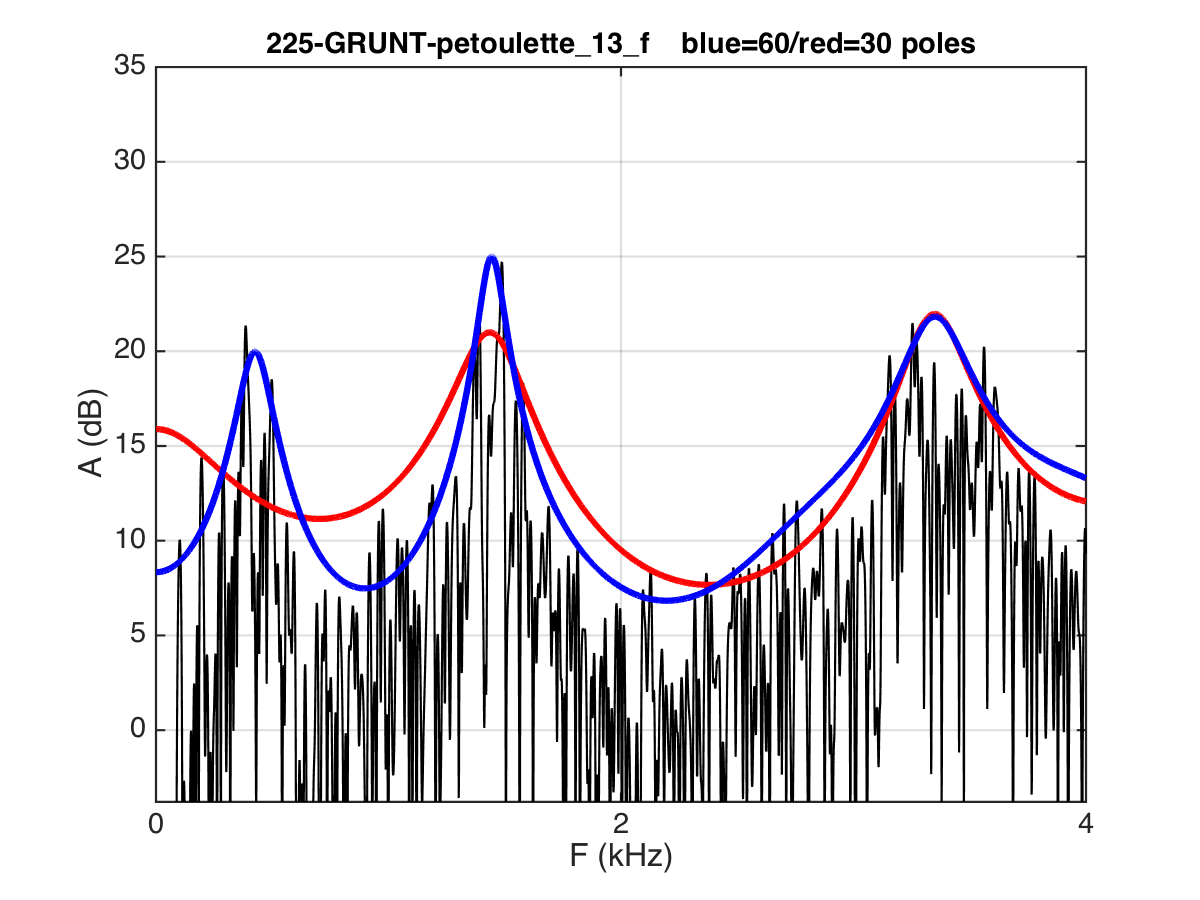

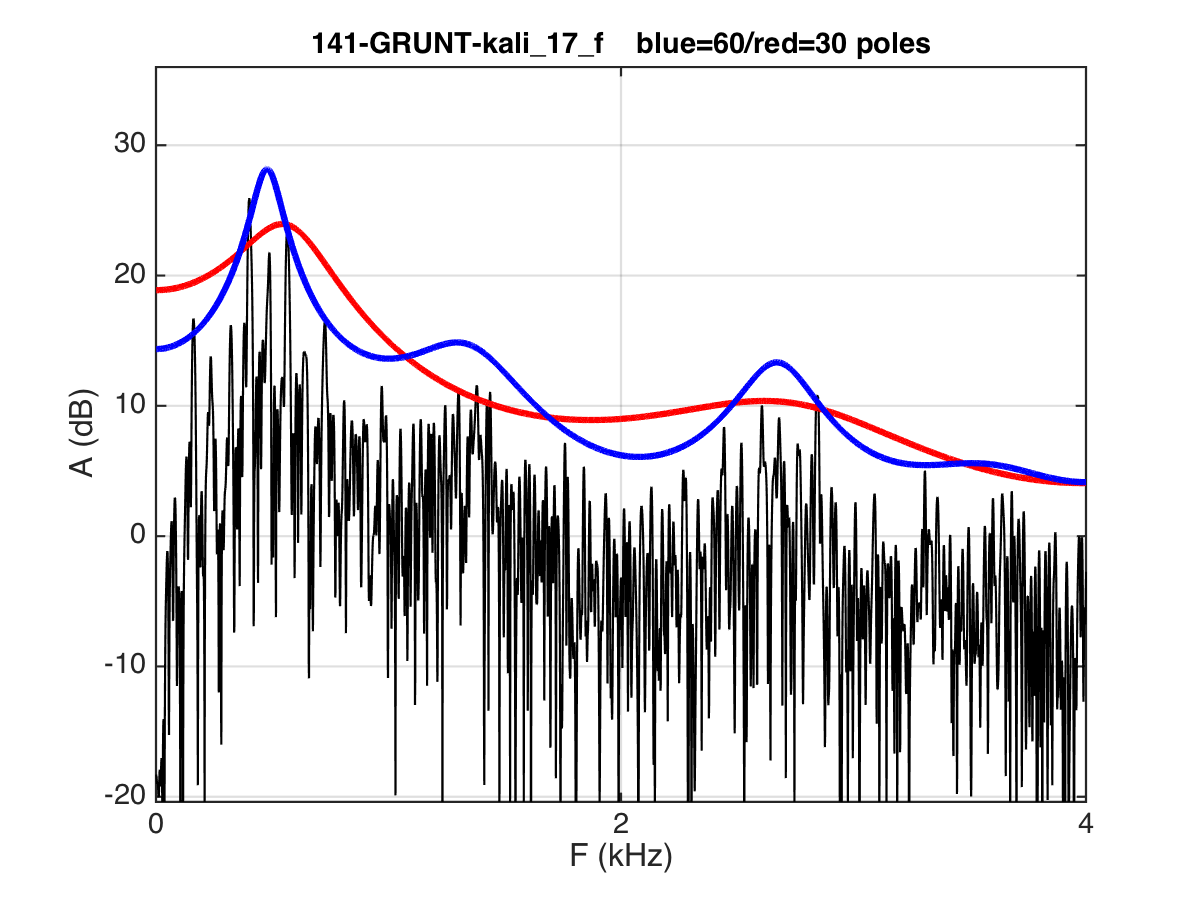


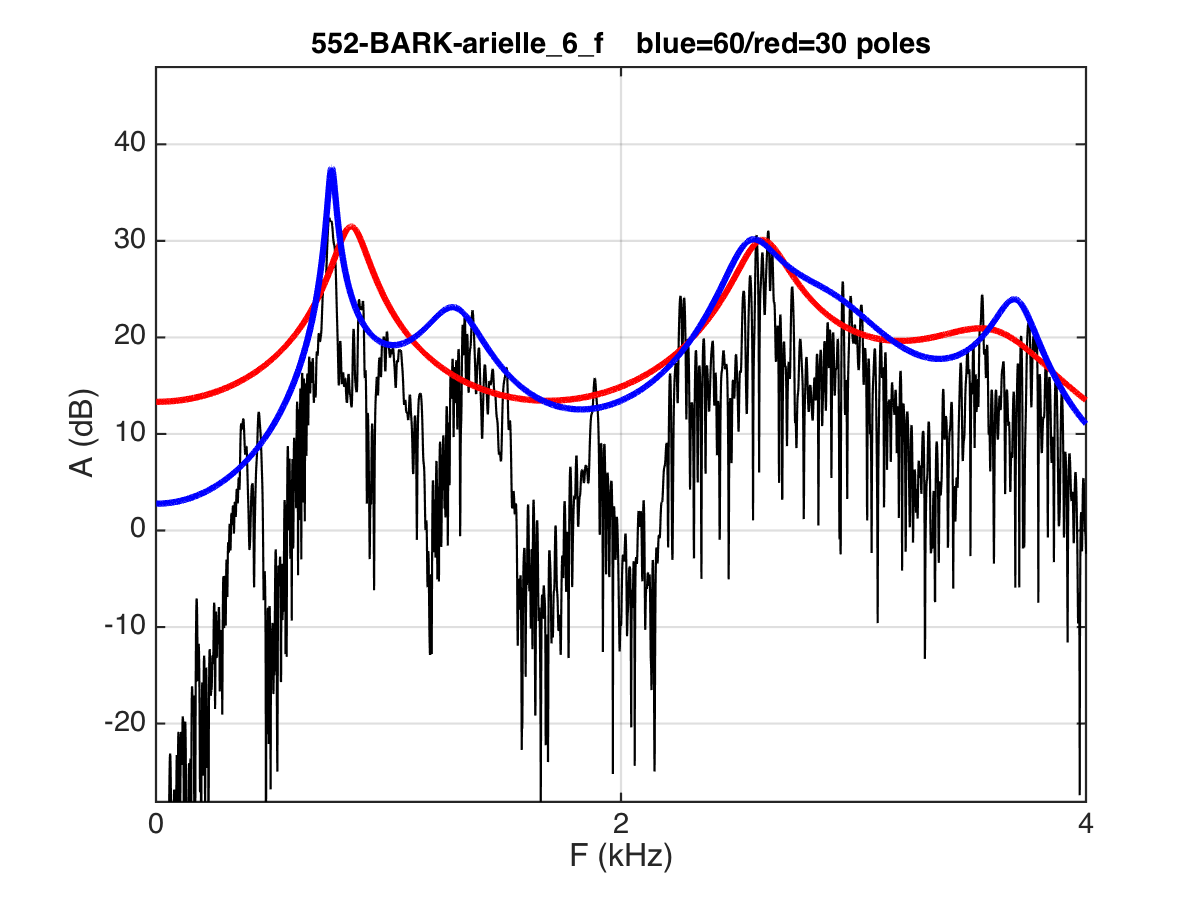

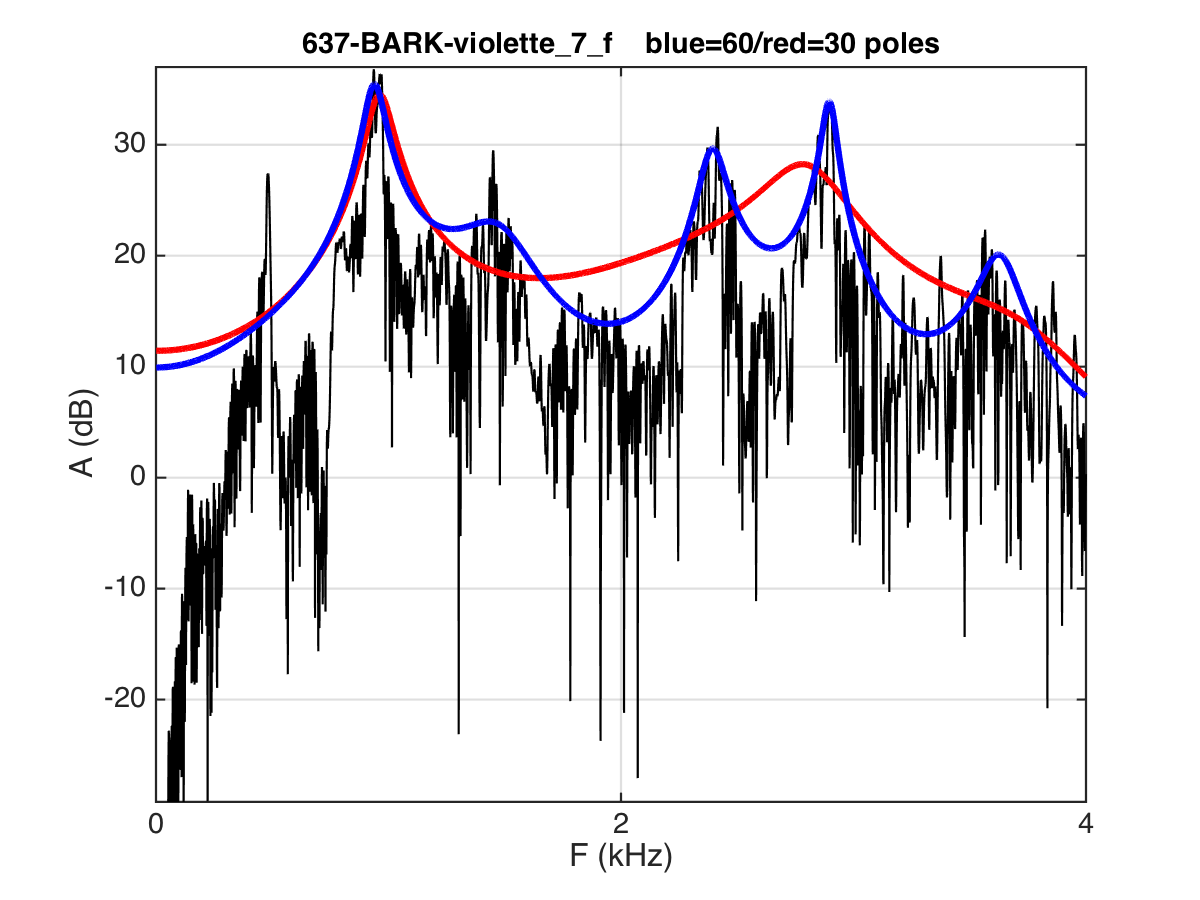


Figure S1 Using pole settings to avoid LPC formant detection errors. Example LPC analyses of two grunts (top) and two barks (bottom), with 30 poles (red) and 60 poles (blue) superimposed on an FFT analysis. Both LPC & FFT calculated using MATLAB. For the grunts (F0 low) only the LPC with 60 poles fits the FFT well. LPC with 30 poles misses the first formant in the left grunt and the second formant in the grunt on the right. On the other hand, for the barks (F0 high) the FFT is well fitted with 30 poles and the formants are well detected. With 60 poles, spurious peaks related to harmonics are erroneously detected.

| _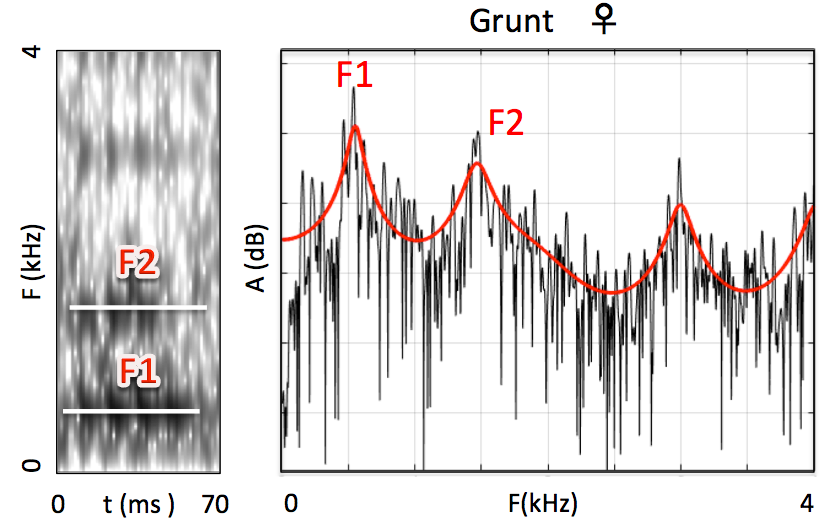_ | 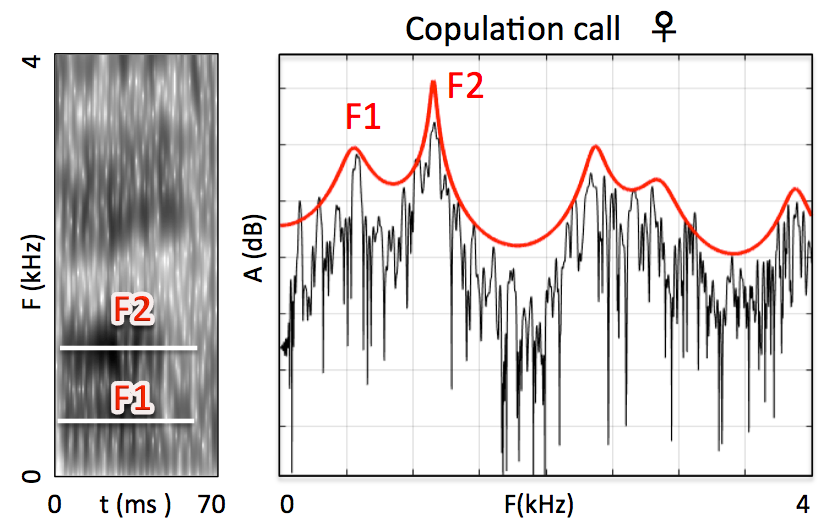 |
| --- | --- |
| 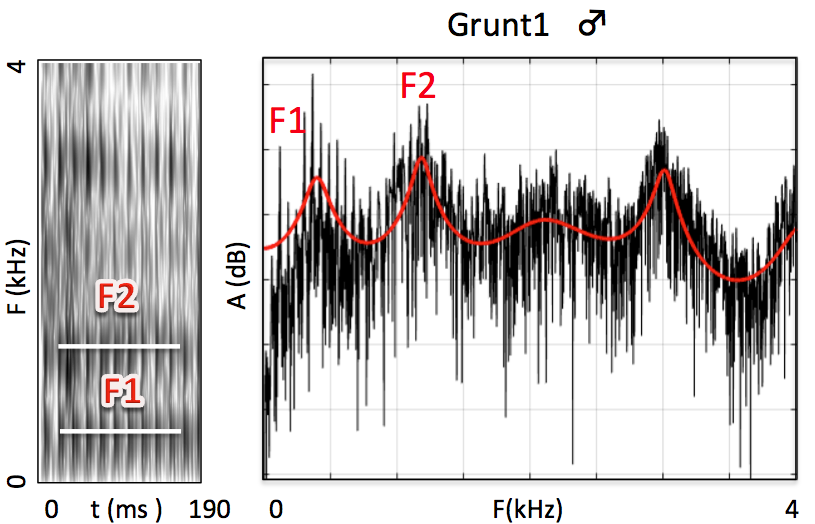 | 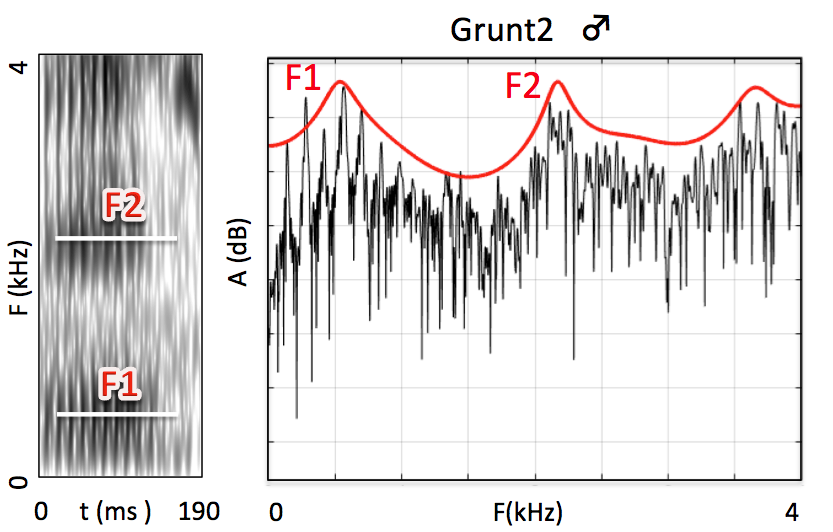 |
| 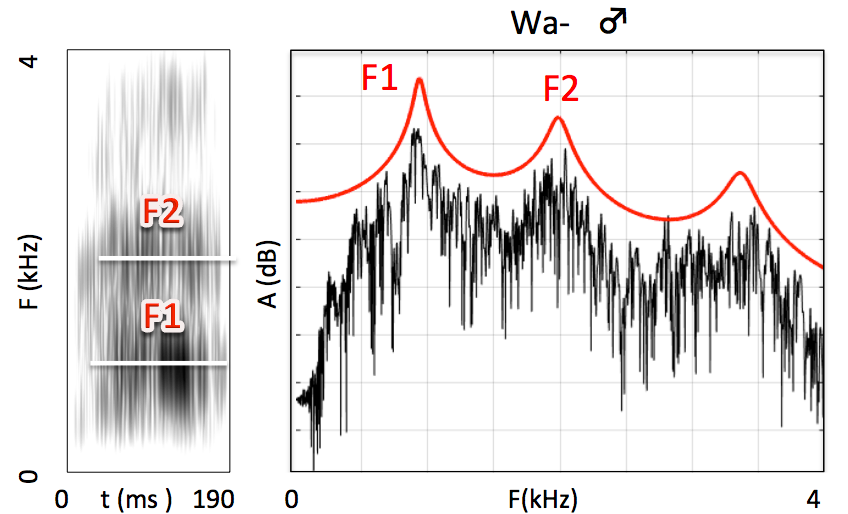 | 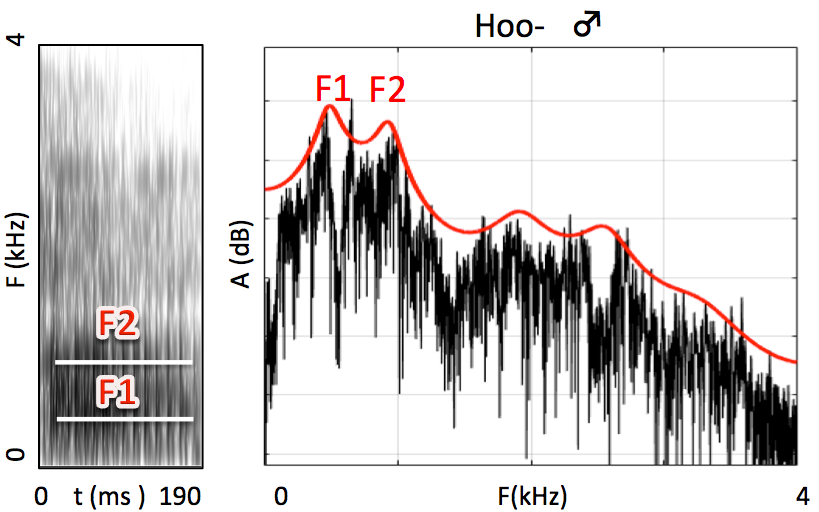 |
| 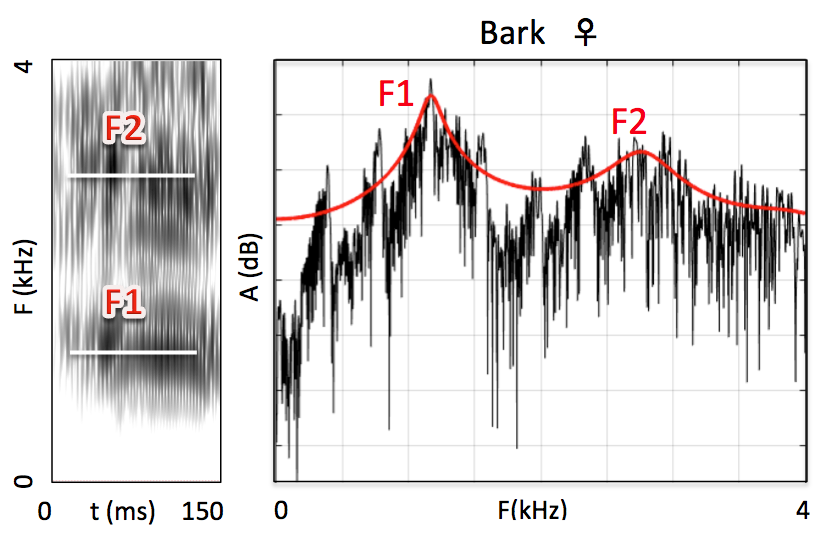 | 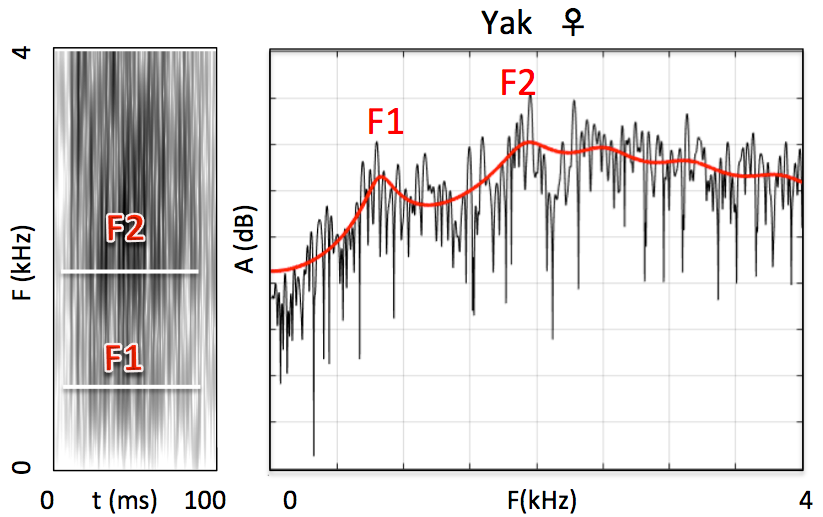 |

Fig S2. Examples of spectrograms (from Praat, available at <http://www.fon.hum.uva.nl/praat/>) and overlaid FFT and LPC spectra (calculated using MATLAB) for grunts (♀♂), copulations calls (♀), wa- (♂), -hoo(♂), barks (♀), yaks (♀). (LPC was set to 60 poles for grunts, copulations calls (♀),-hoo(♀) and yaks, 30 poles for barks, and wa-. Sampling frequency was 44.1 kHz.


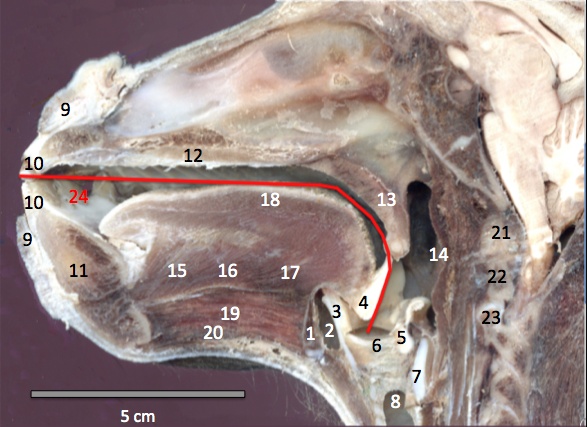


**Figure S3**. Anatomic sagittal view of the head of a female baboon: (1) hyoid bone, (2) air sac, (3) thyroid cartilage, (4) epiglottis, (5) arytenoid cartilage, (6) vocal folds and glottis, (7) cricoid cartilage, (8) trachea, (9) lips, (10) incisors, (11) mandible, (12) hard palate, (13) velum, (14) pharyngeal wall, (15-16-17) anterior GGa, medial GGm, and posterior genioglossus GGp,(18) superior longitudinalis, (19) geniohyoid GH, (20) digastric anterior, (21) C1, (22) C2,(23) C3, (24) mid sagittal line of the vocal tract used to infer the tract length and the computation of the MAS. Note the orientation of the fibers of the GGa, GGm and GGp muscles, which approach vertical on the anterior part of the tongue but are effectively horizontal in the posterior part. The fibers of the styloglossus (SG) muscle on the lateral sides of the tongue have approximately the same inclination as those of a human baby [10]. As in humans, the hyoglossus (HG) muscle has two components which are inserted into the body of the hyoid bone and over the entire extent of the great horn. Its fibers are oriented vertically as found in human children. (N.B.: SG and HG are both lateral to the midline, and do not appear on this view.) This anatomical study shows that a baboon’s tongue has the same musculature as a human’s. Regarding shape and proportions, the baboon’s tongue is more similar to that of a child than that of a human adult.

**Supplemental References**

1. Chiba T, Kajiyama M. The vowel: its nature and structure. Tokyo-Kaiseikan; 1941.

2. Fant G. Acoustic theory of speech production: with calculations based on x-ray studies of Russian articulations. ’s-Gravenhage: Mouton and Co.; 1960.

3. Lieberman PH. Vocal tract anatomy and the neural bases of talking. J Phon. 2012;40: 608–622. doi:10.1016/j.wocn.2012.04.001

4. Boë L-J, Badin P, Ménard L, Captier G, Davis B, MacNeilage P, et al. Anatomy and control of the developing human vocal tract: a response to Lieberman. J Phon. 2013;41: 379–392. doi:10.1016/j.wocn.2013.04.001

5. International Phonetic Association. Handbook of the International Phonetic Association: a guide to the use of the International Phonetic Alphabet. Cambridge, U.K.; New York, NY: Cambridge University Press; 1999.

6. Lee S, Potamianos A, Narayanan S. Acoustics of children’s speech: developmental changes of temporal and spectral parameters. J Acoust Soc Am. 1999;105: 1455–1468. doi:10.1121/1.426686

7. Goldstein UG. An articulatory model for the vocal tracts of growing children [Internet]. Doctoral Thesis, Massachusetts Institute of Technology. 1980. Available: http://dspace.mit.edu/handle/1721.1/16118

8. Rendall D, Kollias S, Ney C, Lloyd P. Pitch (F0) and formant profiles of human vowels and vowel-like baboon grunts: the role of vocalizer body size and voice-acoustic allometry. J Acoust Soc Am. 2005;117: 944–955. doi:10.1121/1.1848011

9. Hall KR, DeVore I. Baboon social behavior. In: DeVore I, editor. Primate behavior: field studies of monkeys and apes. New York: Holt, Rinehart and Winston; 1965. pp. 53–110.

10. Denny M, McGowan RS. Implications of peripheral muscular and anatomical development for the acquisition of lingual control for speech production: a review. Folia Phoniatr Logop. 2012;64: 105–115. doi:10.1159/000338611
